# Digital Health Resilience and Well-Being Interventions for Military Members, Veterans, and Public Safety Personnel: Environmental Scan and Quality Review

**DOI:** 10.2196/64098

**Published:** 2025-04-01

**Authors:** Rashell R Allen, Myrah A Malik, Carley Aquin, Lucijana Herceg, Suzette Brémault-Phillips, Phillip R Sevigny

**Affiliations:** 1 School and Clinical Child Psychology Faculty of Education University of Alberta Edmonton, AB Canada; 2 Heroes in Mind, Advocacy and Research Consortium Edmonton, AB Canada; 3 Department of Occupational Therapy Faculty of Rehabilitation Medicine University of Alberta Edmonton, AB Canada; 4 Counselling Psychology Faculty of Education University of Alberta Edmonton, AB Canada

**Keywords:** public safety personnel, veteran, military member, web-based program, resources, resilience, mobile app, quality review, well-being, military, environmental, review

## Abstract

**Background:**

Accessible mental health care, delivered via mobile apps or web-based services, may be essential for military members, public safety personnel (PSP), and veterans, as they report numerous barriers to seeking in-person care and are at an increased risk for a number of psychological disorders.

**Objective:**

We aimed to identify, describe, and evaluate apps, resource banks (RBs), and web-based programs (WBPs), referred to as digital mental health interventions (DMHIs), recommended for military members, PSP, and veterans. A multidimensional and multisystemic view of resilience and well-being were maintained throughout this environmental scan.

**Methods:**

Information was gathered from a comprehensive review of peer-reviewed literature, a Google search, and a targeted search of websites relevant to the study populations. DMHIs aimed at supporting resilience or well-being were included in the review, including those published in peer-reviewed articles, and those offered to these populations without research or literature backing their use.

**Results:**

In total, 69 DMHIs were identified in this study, including 42 apps, 19 RBs, and 8 WBPs, and were described based on 3 questions related to purpose, strategies, and evidence from the adapted Mobile App Rating Scale and the Mobile App Rating Scale. Each WBP and RB was then reviewed via the adapted Mobile App Rating Scale and each app via the Alberta Rating Index for Apps (ARIA). Overall, 24 (35%) of the DMHIs were recommended for military members, 20 (29%) for PSP, and 41 (59%) for veterans. The most common aim across apps, RBs, and WBPs was to increase happiness and well-being, and the most common strategies were advice, tips, and skills training. In total, 2 apps recommended for military members—PTSD Coach and Virtual Hope Box—received a high rating on the ARIA subscales and have also been trialed in pilot randomized control trial (RCT) and RCT evaluations, respectively, with positive initial results. Similarly, 2 apps recommended for PSP—PeerConnect and R2MR—have been trialed in non-RCT studies, with partially positive outcomes or little to no contradictory evidence and received a high rating on the ARIA. Finally, 2 apps recommended for veteran populations—PTSD Coach and VetChange—received high ratings on the ARIA and have been trialed via pilot-RCT and RCT studies, respectively, with positive outcomes.

**Conclusions:**

In conclusion, there is a need for efficacy and effectiveness trials for DMHIs for military members, PSP, and veterans to ensure that they are effectively meeting the population’s needs. While there appears to be many promising DMHIs, further research is needed before these interventions continue to be promoted as effective and widely distributed.

## Introduction

### Background

Military members (eg, Canadian Armed Forces, sailors, soldiers, aviators, and special forces members) and public safety personnel (PSP), or a person who fulfills duties related to public safety [1], show extraordinary commitment to the service and care of Canadians. These individuals, including those involved in emergency response, disaster relief, and national security, are our most critical defenses. Their positions require that they remain operationally ready to respond at a moment’s notice to local, provincial, national, and international needs. Similarly, veterans, or those who have served with the military and have transitioned to civilian life, must remain supported after their service. The purpose of this paper is to complete an environmental scan to summarize key information and review the quality of resilience and well-being app- and web-based programming for military members, PSP, and veterans. To do so, population characteristics, the barriers they experience accessing mental health care, and the current landscape of mental health mobile apps, resource banks (RBs), and web-based programs (WBPs), hereafter referred to as digital mental health interventions (DMHIs), must be considered.

### Population Characteristics

Occupational duties conducted by military members, PSP, and veterans have several implications on their health and well-being. This can include exposure to potentially psychologically traumatic events (PPTEs), such as sudden violent death, sudden accidental death, serious transportation accident, and physical assault [2,3]. Carleton et al [3] reported that, for PSP, exposure to PPTEs was associated with positive screens for a variety of psychological disorders, including posttraumatic stress disorder (PTSD), depression, anxiety, and substance use.

In addition, military members are at increased risk for exposure to PPTEs [4]. Notably, Iraq and Afghanistan war veterans are likely to report lifetime PTSD, substance use disorders, and nonsuicidal self-injurious behaviors, as well as current alcohol use disorder, substance use disorder, and suicidal ideation [5]. In a population-based sample of US veterans, 86.12% (2719/3157) reported exposure to at least one PPTE, with a mean of 3.4 (SD 2.8) different PPTEs in their lifetime [6]. The number of PPTEs experienced have been shown to exacerbate PTSD symptoms, which is related to greater cognitive difficulties, increased loneliness, and lower functional social support [7]. Understanding occupational and operational PPTEs and their impact is critical when working with military members, PSP, and veterans.

### Barriers to Mental Health Care

Access to care is essential for populations that experience an increased risk of exposure to PPTEs and mental and physical health difficulties. There are several barriers to accessing and seeking care at both the individual and systems levels. From a strictly physical perspective, Canada is a vast country and access to services may vary depending upon where one lives. For example, while there may be a multitude of service options available in large urban settings, the same is not necessarily true for those living in rural or remote areas. On a psychological level, stigma is one of the most common barriers for military members, PSP, and veterans seeking care. PSP often attribute perceived or experienced stigma as a barrier not only because of the psychological disorder itself but because of care-seeking and the cause of the injury, complicating the notion of when care-seeking is acceptable [8]. Some PSP view individuals who are seeking support as “claiming” their mental injuries and “milking the system” for unwarranted personal gain, burdening their colleagues, organizations, and taxpayers by seeking care or speaking up about their mental health difficulties [8].

Stigma is also a perceived barrier to care for veteran and military member populations [4,9,10]. A population-based sample of US veterans found that only 12% reported engagement in mental health care [10]. Of 4069 veterans sampled, 924 (22.7%) met criteria for 1 or more psychological disorders, with 73.1% (675/924) of these individuals reporting no current engagement in treatment [10]. These data are similar for military members, with estimates of 23% to 40% of individuals who screened positive for a mental disorder seeking care [4]. Those who met criteria for a mental disorder were found to be twice as likely to report concern for possible stigmatization as a barrier to seeking care [4]. Some key stigma-related barriers to seeking care identified by military member respondents who met criteria for a mental disorder included: the belief that they will be seen as weak, belief that their unit leadership might treat them differently, and belief it would cause members of their unit to have less confidence in them [4]. Hoge et al [4] found that 55.1% (354/642) of military members in their sample who met the criteria for a mental disorder believed they would have difficulty getting time off work for treatment. Those with negative beliefs about mental health care are also less likely to seek care for their mental health needs [9]. Given that military members, PSP, and veterans are at increased risk of PTSD, mood, anxiety, and substance use disorders [3,6,11], it is paramount to consider factors or alternative treatment modalities that increase their access to support and services.

### Well-Being and Resilience

A holistic well-being approach to working with these populations is essential considering the importance of well-being on a person’s ability to adapt to stress and PPTEs [12]. Research has shown that the domains of well-being interact and affect other areas of well-being, as well as impact one's resilience response [12,13]. Our current review maintains a multidimensional perspective of well-being, which considers the degree to which individual, dyadic, community, and organizations’ needs are satisfied [14,15]. For example, enhanced community well-being has been shown to lead to an increased likelihood of a resilience response [16,17] and has potential positive effects across other domains of well-being and systems surrounding the individual [18,19].

Access to holistic care is essential to promoting resilience. Resilience is understood by the authors to be a system’s process of adaptation (eg, individuals, groups, and organizations) following adversity or risk exposure. This multisystemic perspective views resilience as a dynamic process influenced by socioecological system interactions, available resources, and individual qualities, which function as protective factors and increase the likelihood of coping with and overcoming adversity [20-22].

### Background on DMHIs

Asynchronous interventions, available on the web or via mobile phone, may offer increased access to care and potentially decrease military members’, PSP, and veterans’ experience of stigma. In response to the COVID-19 pandemic, there was a notable increase in the development of technologies in response to the needs of the public [23]. Digitally delivered mental health interventions via the internet or mobile app are relevant for purposes of this paper. Most internet-based interventions are psychological treatments that are disorder-specific and are typically grounded in cognitive behavioral therapy (CBT) [24]. Potential benefits of asynchronous self-guided internet-based interventions include that they are easily accessible, anonymous, and may reach populations that may not otherwise seek treatment [24]. A systematic review and meta-analysis (k=11, 12 comparisons) by Díaz-García et al [25] reported nonsignificant changes in direct and proximal resilience measures for internet-based interventions (effect size for resilience was g=0.12, *P*=.32). Kuester et al [24] conversely reported that internet-based interventions using a cognitive-behavioral therapy framework (k=7) were effective compared to passive control conditions in reducing PTSD symptoms (g=0.72, *P*<.001). When compared to active control conditions, there was no significant difference for changes in PTSD symptoms [24]. Further research is yet needed to further evaluate such programs.

Mobile apps appear to be a less effective form of treatment modality. In a review of meta-analyses, pooled effect sizes across 4 meta-analyses reported small-to-moderate results (g=0.28-0.38), with the small or nonsignificant effect sizes for intervention versus active controls (g=0.17-0.21) [23]. A meta-analysis found that smartphone interventions for depression (k=6, g=0.33, *P*=.005), anxiety (k=6, g=0.30, *P*=.15) suicidal ideation (k=4, g=–0.14, *P*=.25), self-injurious behaviors (k=3, g=–0.04, *P*=.75), smoking (k=3, g=0.39, *P*.001), drinking (k=3, g=–0.03, *P*=.77), sleep problems (k=2, nonpooled effects g=0.72 and 0.84, *P*<.05), and PTSD (k=2, g=–0.05 and 0.15, *P*>.05) resulted in small or nonsignificant changes compared to controls [26]. Caution was also noted for the moderate to high heterogeneity between trials for anxiety, depression, and substance use (smoking and drinking) [26]. Donker et al [27] also highlighted mixed results in terms of 5 mobile apps targeting depression, anxiety, and substance use. Due to the quality of these studies and the risk of bias, Donker et al [27] cautioned against the results reported by these studies. Overall, the within-group effect sizes ranged from –0.45 to 2.28 following the intervention, and 0.45-2.11 after follow-up. Some mobile apps were compared to control groups (ie, a CBT computer program, attentional control, and attentional control plus data summaries and meeting with general practitioner), and the between group effects ranged from –0.14 to 0.25 following the intervention, and –0.28 to 0.58 after follow-up [27]. Finally, a review of mental health mobile apps found that there is little evidence to suggest that mobile-based interventions are helpful, and that some are harmful [28].

Several limitations have been reported regarding app use to address mental health concerns. Many mobile apps have not been subjected to research validation and have privacy and confidentiality concerns [29], with evidence suggesting that the effectiveness and efficacy of mobile apps are questionable [23,26-29]. Skorburg and Yam [23] highlighted further concerns that the apps may exacerbate health inequalities. Veterans living in rural locations particularly report that apps are hard to navigate, and their use is impacted by financial and connectivity limitations [30]. Attitudes of app use in veteran populations also appear extreme, being either strongly positive or strongly negative [30]. Lack of awareness of the apps and low rate of veteran app use was also noted to be common [31].

### Objectives

This environmental scan aimed to provide a review and quality assessment of DMHIs, including apps (ie, mobile apps), RBs (ie, websites with resources and information), and WBPs (ie, interactive programs available on the web), recommended for military members, PSP, and veterans. Apps and WBPs are similar in that they are interactive resources that may include modules, questionnaires, audio or video information, and intervention-specific activities. RBs may not be interactive in nature, but they were included in this review as they provide the target populations with valuable information related to resilience and well-being. In addition, the review was meant to be as inclusive as possible in identifying resources available to these populations and assessing the quality of such resources. Therefore, all available resources for the target populations, including apps, RBs, and WBPs, were examined.

Environmental scanning is the acquisition and use of information about trends and relationships in the environment to determine information needs and use [32]. The objectives of this project were to (1) conduct an environmental scan of well-being and resilience DMHIs available in Canada for military members, PSP, and veterans and (2) review the quality of the available programs. Information was gathered through an iterative search of peer-reviewed literature (ie, a scoping review search), a Google search, and a targeted search of websites relevant to the study populations. As DMHIs are widely available and accessible, regardless of the evidence to support their use, it is essential to review all DMHIs to evaluate their quality, including usability, privacy, functionality, and information quality. DMHIs vary in terms of their interactive or static nature of their delivery format. Therefore, we aimed to assess both the DMHIs that are more interactive in nature (ie, apps and programs) and those that are more static (ie, resources). Therefore, this environmental scan will add to the academic literature by reviewing DMHIs with and without peer-reviewed literature backing their use and evaluating those that are available in Canada.

## Methods

### Overview

This environmental scan used 3 steps, including app identification, description, and evaluation. Step 1 involved several methodologies to first identify relevant DMHIs: (1) an iterative search of all available peer-reviewed literature, conducted in February 2024; (2) a Google search, conducted in June 2023; and (3) a targeted web search of 12 websites, completed in August 2023 (eg, Veterans Affairs Canada website). Once identified, step 2 included assessing each DMHI via questions on the adapted Mobile App Rating Scale (A-MARS) [33] and the Mobile App Rating Scale (MARS) [34] to evaluate DMHI purpose, strategies, and evidence base. In step 3, each app was evaluated with the Alberta Rating Index for Apps (ARIA) [35], and each RB and WBP was assessed with the A-MARS [33]. While there is no registered protocol for the current project, the information provided in the methods may be used to replicate the current search. A description of the methods associated with each step is provided in subsequent sections.

### Step 1: Identification of DMHIs

#### Search Strategy and Information Sources

The literature search was used to gather key information to determine the depth and breadth of peer-reviewed literature related to DMHIs well-being and resilience resources for military members, PSP, and veterans, with key terms related to (1) population (eg, military members), (2) resilience and well-being related constructs (eg, hardiness), and (3) web- or mobile-based programs (eg, mobile apps; an example of a full search string is provided in Multimedia Appendix 1). The final search was conducted using a Boolean format of the following databases: Academic Search Complete, CINAHL, APA PsychInfo, Embase, SocINDEX, and MEDLINE. The Google search was conducted using key terms based on the same 3 concepts, but because Google limits the characters for each search, multiple searches were conducted (full search string is provided in Multimedia Appendix 2). Finally, the targeted website search included websites developed for each population (eg, Veterans Affairs), and each website was thoroughly searched for any well-being or resilience resources recommended for their members (the list of targeted websites is provided in Multimedia Appendix 3).

#### Eligibility Criteria

Apps, RBs, and WBPs included in this study encompassed resources that were aimed at supporting resilience and well-being in military members, PSP, and veterans (Textbox 1). While the focus of our study was to review resources relevant to military members, PSP, and veterans, DMHIs developed for and tested with the general population were incorporated if they were also recommended for (but not necessarily trialed with) one of these populations. In total, 4 researchers (RRA, MAM, CA, and LH) were involved in the eligibility assessment and selection process. Only DMHIs that were free (or had free components), available in Canada, and available on Apple, Google Play, and on the web without enrollment access met the criteria. At all levels of screening, a minimum of 2 researchers reviewed each app and website independently. All apps, programs, and websites with discrepant ratings were reviewed independently by a third reviewer. If necessary, the resource was discussed in a research team meeting before final eligibility decisions were made. Each DMHI was grouped by type of resource (ie, app, RB, or WBP) for the remaining steps: description and quality assessment.

**Eligibility criteria for digital mental health interventions (DMHIs).**

**Inclusion criteria**

- Self-directed DMHIs meant to improve well-being or resilience
- The DMHI was recommended for or developed to support military members, public safety personnel, or veteran populations
- DMHI was available on the Apple or the Google Play Store or free to access on the web (or some features were accessible at no cost)

**Exclusion criteria**

- The DMHI included a guided table 1(synchronous) support component for the intervention (eg, in-person or virtual check-ins [or sessions], therapist interaction, virtual reality, or in-person meetings or discussions)
- DMHI was not available in the English language or not available in Canada
- DMHI required enrollment into the program or access code

### Step 2: Description of DMHIs

Once eligibility was determined, each DMHI was reviewed and data were extracted and recorded by a minimum of 2 researchers to ensure relevant information was included in the review (eg, the description of the resource and the population the resource was developed, trialed, tested, or recommended for). With environmental scans being largely descriptive in nature and aimed at capturing the current state of the literature, the authors collected information about the type of research available (ie, no research and level of evidence), as well as the focus and strategy of each DMHI. These were then used to describe each DMHI and to describe the intervention focus, the strategies commonly used, and the overall state of the evidence.

The classification section of the MARS [34] was used to describe the focus and strategies used by each DMHI. The focus question evaluated what the intervention targeted and included 13 items in which the researchers selected all that applied, including whether the DMHI targeted (1) to increase happiness or well-being, (2) mindfulness or meditation or relaxation, (3) to reduce negative emotions, (4) depression, (5) anxiety or stress, (6) anger, (7) behavior change, (8) alcohol or substance use, (9) goal setting, (10) entertainment, (11) relationships, (12) physical health, and (13) other (specify). Next, the researchers selected all that applied in terms of the theoretical background and strategies used by each app, RB, and WBP, including (1) assessment, (2) feedback, (3) information or education, (4) monitoring or tracking, (5) goal setting, (6) advice or tips or strategies or skills training, (7) CBT—behavioral, (8) CBT—cognitive, (9) acceptance and commitment therapy, (10) mindfulness or meditation, (11) relaxation, (12) gratitude, (13) strengths-based, and (14) other (specify).

A question on the A-MARS [33] related to evidence base was used to describe the depth and breadth of the evidence for all DMHIs. Each DMHI was summarized based on this question to determine whether the app or electronic tool (e-tool) had been trialed or tested and verified by evidence in published scientific literature. Each app, RB, and WBP was classified into 1 of 6 groups based on their evidence in the literature:

- It has not been trialed or tested.
- The evidence suggests the app or e-tool does not work.
- App or e-tool has been trialed (eg, acceptability, usability, and satisfaction ratings) and has partially positive outcomes in studies that are not randomized controlled trials (RCTs), or there is little or no contradictory evidence.
- App or e-tool has been trialed (eg, acceptability, usability, and satisfaction ratings) and has positive outcomes in studies that are not RCTs, and there is no contradictory evidence.
- App or e-tool has been trialed and outcome tested in 1 to 2 RCTs indicating positive results.
- App or e-tool has been trialed and outcome tested in >3 high quality RCTs indicating positive results.

### Step 3: Evaluation of DMHIs

Each DMHI was evaluated based on 2 quality rating scales: the A-MARS and the ARIA. A description of each is provided in the subsequent sections.

#### Adapted MARS: RBs and WBPs

The A-MARS [33] is a rating scale adapted from the MARS [34] and was used to review RBs and WBPs. The A-MARS was developed to evaluate health-related e-tools, with a specific expansion of the engagement subscale. The A-MARS is a 29-item scale rated on a scale from 1 (*inadequate*) to 5 (*excellent*), with the following subscales: engagement (5 items), functionality (4 items), aesthetic (3 items), information (6 items), subjective quality (4 items) and health-related quality (6 items). The subscale items were summed and averaged for a subscale score, then the engagement, functionality, esthetics, and information subscales were summed and averaged for a mean quality score, and finally, all subscales were summed and averaged for an overall mean total score. The A-MARS total mean score is a reflection of the overall quality of the e-tool, whereas the subscale scores and mean quality score describe the specific strengths and weaknesses of the e-tool.

For all subscales of the A-MARS, intraclass correlation coefficient (ICC) estimates and their 95% CIs were calculated using SPSS statistics (version 29; IBM Corp) based on a mean rating (k=2), absolute-agreement, 2-way mixed-effects model. For RBs, the reliability of raters fell in the excellent range (ie, ICC>0.90), with the exception of the aesthetic domain (ICC=0.73; 95% CI 0.33-0.90) [36]. For WBPs, the reliability of raters fell in the excellent range (ICC>0.90) for all domains [36].

#### The ARIA: Apps

The ARIA [35] served as a measure to evaluate the quality of eligible apps. For this study, the care provider version was used, with 2 sections: part A and part B. Part A was completed before downloading the app and was based on the information on the app store page. Part A was used to assess goal fit, trustworthiness, privacy, and affordability. Next, part B was completed after 2 researchers used the app independently for at least 10 minutes to assess quality related to security, trustworthiness, ease of use, functionality, fit for population, usefulness, and satisfaction [37]. The original ARIA scale was on a scale from 0 to 4, but for the purpose of this review, and to remain consistent with the A-MARS, the scale was changed so each item in part A and part B were rated on a scale of 1 (*strongly disagree*) to 5 (*strongly agree*). Subjective quality of each app was also assessed, and these items included “I would recommend using this app to the user” and “the number of stars that best represents your overall rating for the quality of this app is,” on a scale from 1 (*strongly disagree; 1 star, worst app*) to 5 (*strongly agree; 5 stars, best app I have every used*).

After completing part A and B, the scores for each item were added up for a total score out of 30 for part A, and 60 for part B. A higher score in part A indicates that the app fits the purpose of the project, is trustworthy, has adequate privacy, and is affordable [37]. A higher score in part B indicates better quality of content and usability of the app [37]. ICC estimates and their 95% CIs were calculated using SPSS statistical package version 29 based on a mean rating (k=2), absolute-agreement, 2-way mixed-effects model. The ICC for part A (ICC=0.86; 95% CI 0.75-0.92) and part B (ICC=0.51; 95% CI 0.07-0.75) indicate good and moderate reliability across raters, respectively [36].

## Results

### Step 1: Identification of DMHIs

#### Literature Review Article Identification

**Figure 1:**
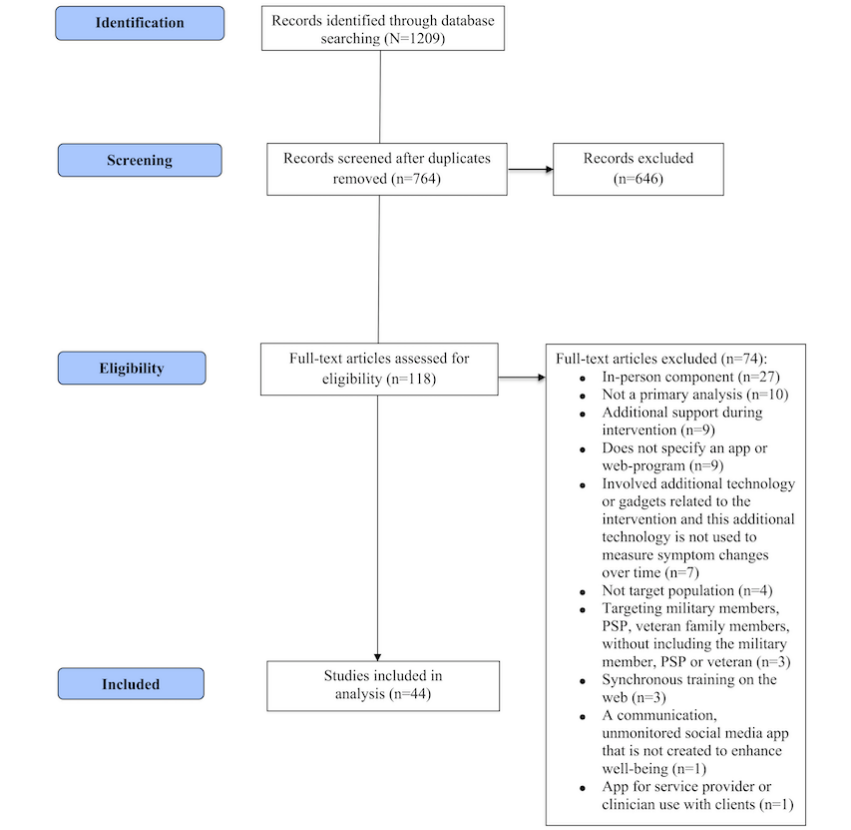
PRISMA-ScR (Preferred Reporting Items for Systematic Reviews and Meta-Analyses Extension for Scoping Reviews) flowchart. PSP: public safety personnel.

The initial review of the literature yielded a total of 1209 articles, with 764 duplicates, resulting in 646 articles. In total 3 researchers (RRA, MAM, and CA) completed initial reviews of titles and abstracts identified during the literature review, with a range of agreement (Cohen k) from moderate (k=0.48) to substantial (k=0.79). The authors included all articles with disagreement in the full-text review to allow a more comprehensive evaluation of the discrepancies. A total of 118 articles met the criteria for full-text review. The agreement for full-text reviews was fair (k=0.20) to substantial (k=0.79). In the end, 44 articles were included for the DMHI screening. Figure 1 illustrates the PRISMA-ScR (Preferred Reporting Items for Systematic Reviews and Meta-Analyses Extension for Scoping Reviews) literature review flowchart.

#### DMHI Identification

**Figure 2:**
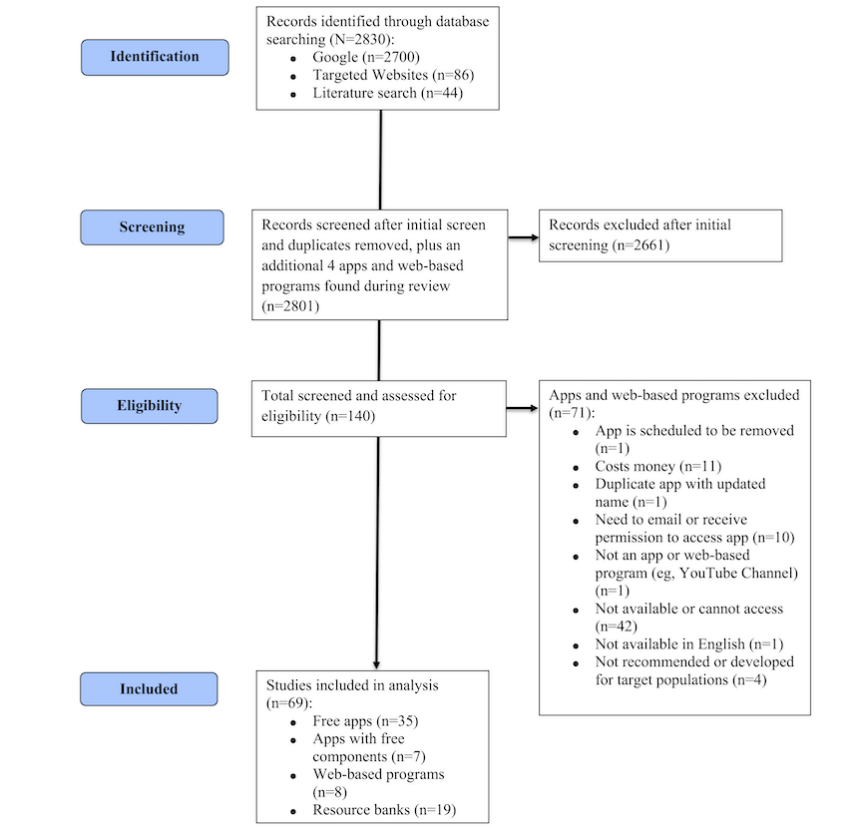
Environmental scan flowchart.

To review DMHIs available to the target populations in Canada, a comprehensive Google search and targeted web search were also conducted. In the end, the literature search yielded 44 digital wellness resources, the Google search yielded a total of 2700 Google records, and the targeted web search yielded 12 websites with 86 digital wellness resources. Each DMHI and resource was reviewed by a minimum of 2 reviewers, and discrepancies were rectified by a third reviewer or group discussion (authors involved in review included RRA, MAM, CA, and LH). Details of the review and screening process for accessible DMHIs are provided in the PRISMA (Preferred Reporting Items for Systematic Reviews and Meta-Analyses) flowchart (Figure 2). After the initial screening of the literature search, Google search, and targeted web search, 140 apps, RBs, and WBPs were identified for further review. In total, 2 authors (RRA and MAM) conducted a further review to determine if the app or website was accessible and available (ie, available in Canada, free [or free components], and no sign-up required). A total of 69 relevant digital wellness programs were identified and included in this study, including 42 apps (7 apps with free components), 8 WBPs, and 19 web-based RBs.

### Step 2: Description of DMHIs

The following results are a descriptive summary of all information gathered via the A-MARS and MARS. Key information was also recorded for each included program (eg, population recommended for) and is summarized in subsequent sections.

#### Characteristics of DMHIs

##### Overview

Most of the programs (41/69, 59%) focused on targeting veteran populations, followed by 35% (24/69) of programs recommended for military members, and 29% (20/69) of programs recommended for PSP. A small portion of programs (2/69, 3%) were recommended for military member and veteran families (Table 1). The aim of this project was to assess DMHIs available to military members, PSP, and veteran populations in Canada. Although a majority of DMHIs were developed outside of Canada, with 10% (7/69) DMHIs originating in Canada, the information, resources, tools, and activities provided by the DMHIs appeared to transcend national borders. In addition, it appeared that all DMHIs were developed without targeting a specific sex or gender, or it was unclear whether the intervention was developed for a specific gender.

**Table 1:** Digital mental health interventions key information.

Digital mental health resource name	Type of resource	Location the digital mental health intervention was created	Suggested population
7-Minute Chi^a,b,c^	App	Belgium	Veterans
AboutFace^c^	Resource bank	United States	Veterans
ACT Coach^d^	App	United States	Veterans
AIMS for Anger Management^c^	App	United States	Military members and veterans
AIMS for Anger Management^e^	Web-based program	United States	Military members and veterans
Beyond MST^b,c^	App	United States	Military members and veterans
Breathe2Relax^a,e^	App	United States	Military members
Calm^b,e^	App	United States	Public safety personnel
CBT-Insomnia Coach^e^	App	United States	Veterans
Chill Drills^c^	App	United States	Military members and veterans
Chris Germer Meditations^c^	Resource bank	United States	Veterans
Comfort Talk Pro^e^	App	United States	Veterans
Couples Coach^e^	App	United States	Veterans
COVID Coach^e^	App	United States	Military member, PSP, and veterans
CPT Coach^e^	App	United States	Veterans
CrewCare^c^	App	United States	Public safety personnel (fire)
Daily Yoga^b,e^	App	China	Military members
Drinks:Ration^a,f^	App	United Kingdom	Military members and veterans
Driven Resilience^b,c^	App	Australia	Public safety personnel and military member
Equipt^c^	App	Canada	Public safety personnel (police)
eQuoo^a,d^	App	United Kingdom	Veterans
Exalted Warrior Foundation^c^	Resource bank	United States	Veterans
First Responders First^c^	Resource bank	Canada	Public safety personnel
FOCUS on the Go!^a,c^	App	United States	Military member families and veteran families
Freedom Qigong^c^	Web-based program	United Kingdom	Veterans
Head to Health^c^	Resource bank	Australia	Military members
HeadFIT^d^	Web-based program	United Kingdom	Military members
Insight Timer^a,b,d^	App	United States	Public safety personnel
Insomnia Coach^d^	App	United States	Veterans
Lighthouse Health and Wellness^c^	App	United States	Public safety personnel
Manage Stress: VA National Center for Health Promotion and Disease Prevention^c^	Resource bank	United States	Veterans
Meditation Oasis Podcasts^c^	Resource bank	United States	Veterans
Meditation Rx^a,e^	App	United States	Veterans
Mind Resilience^c^	Resource bank	United Kingdom	Public safety personnel
Mindarma^a,c^	App	Australia	Public safety personnel
Mindfulness Coach^d^	App	United States	Public safety personnel and veterans
Mindshift^d,g^	App	Canada	Public safety personnel
Misadventures in Money management^c^	Web-based program	United States	Military members
MOVE! Coach^c^	App	United States	Military members and veterans
National Sleep Foundation^c^	Resource bank	United States	Veterans
NHS Every Mind Matters^c^	Resource bank	United Kingdom	Military members
OSI Connect^e,g^	App	Canada	Veterans
Pain and Opioid Safety^c^	Resource bank	United States	Military members
Pain eHealth for Activity, Skills, and Education^f^	Resource bank	United States	Veterans
PeerConnect^e^	App	United States and Canada	Public safety personnel
PE Coach 2^e^	App	United States	Military members
Provider Resilience^c^	Web-based program	United States	Public safety personnel
PTSD Coach^e^	App	United States	Veterans
PTSD Coach Canada^c^	App	Canada	Military members and veterans
PTSD Family Coach^e^	App	United States	Military members (and families) and veterans (and families)
R2MR^a,e^	App	Canada	Military members, Public safety personnel, and veterans
Responder Strong^c^	Resource bank	United States	Public safety personnel
Shield of Resilience Training^c^	Web-based program	United States	Public safety personnel
Simply Yoga^b,e^	App	United States	Military members
STAIR Coach^e^	App	United States	Veterans
Stand Down: Think Before You Drink^a,b,e^	App	United States	Veterans
Stay Quit Coach^e^	App	United States	Veterans
Substance Abuse and Mental Health Services Administration Disaster App^c^	App	United States	Public safety personnel
SwapMyMood^c^	App	United States	Military members and veterans
Tactical Breather^e^	Web-based program	United States	Military members
Tao Connect^c^	Web-based program	United States	Public safety personnel
Ten Percent Happier^c^	Resource bank	United States	Public safety personnel
VA Make the Connection^c^	Resource bank	United States	Veterans
VA National Center for PTSD^c^	Resource bank	United States	Veterans
VA Public Health^c^	Resource bank	United States	Veterans
VetChange^f^	App	United States	Veterans
Veterans Yoga Project^c^	Resource bank	United States	Veterans
Virtual Hope Box^f^	App	United States	Military members
Yoga Journal^c^	Resource bank	United States	Veterans

^a^Available on Apple App Store only.

^b^App with free component available, but add-ons that cost money

^c^App or electronic tool (e-tool) has not been trialed or tested.

^d^App or e-tool has been trialed (eg, acceptability, usability, and satisfaction ratings) and has positive outcomes in studies that are not randomized controlled trials (RCTs), and there is no contradictory evidence.

^e^App or e-tool has been trialed (eg, acceptability, usability, and satisfaction ratings) and has partially positive outcomes in studies that are not RCTs, or there is little or no contradictory evidence.

^f^App or e-tool has been trialed and outcome tested in 1 to 2 RCTs indicating positive results.

^g^Available on Google Play only.

A summary description of the 8 WBPs, 19 web-based RBs, and 42 apps (7 apps with free components) can be found in the subsequent section.

##### Description of WBPs

Of the 8 WBPs that met the criteria, their purpose varied (the purpose and theoretical orientation for each WBP is provided in Multimedia Appendix 4). Notably, most WBP had multiple areas of focus for both the purpose and theoretical orientation. Therefore, the following totals and percentages represent this overlap and the general representation of each domain across all WBPs. In total, 75% (6/8) of the WBPs focused on increasing happiness and well-being. Next, 50% (4/8) focused on mindfulness, meditation, or relaxation, 38% (3/8) aimed to reduce anxiety and stress, 38% (3/8) focused on reducing negative emotions, and 25% (2/8) aimed to facilitate behavioral change. Finally, a few of the WBPs aimed to support anger management (1/8, 12%), financial management (1/8, 12%), goal setting (1/8, 12%), physical health (1/8, 12%), relationships (1/8, 12%), and resilience (1/8, 12%).

In terms of theoretical background or strategies used, 66% (5/8) of the WBPs focused on advice, tips, strategies, or skills training, 50% (4/8) on information or education, 50% (4/8) on mindfulness or meditation, 38% (3/8) on CBT (behavioral) techniques, 38% (3/8) on CBT (cognitive) techniques, and 38% (3/8) on relaxation strategies. Other theoretical background or strategies highlighted by WBPs included goal setting (2/8, 25%), gratitude modules (2/8, 25%), monitoring or tracking (2/8, 25%), or strengths-based techniques (1/8, 12%). The A-MARS evidence base review demonstrated a lack of evidence for WBPs for military members, PSP, and veterans, with 75% (6/8) of them not being trialed or tested, and only 25% (2/8; AIMS for Anger Management and HeadFIT) of them having been trialed by studies that are not RCTs, with partial positive outcomes, or little contradictory evidence.

##### Description of RBs

Similar to WBPs, each RB had multiple areas of focus for both the purpose and theoretical orientation. The following totals and percentages represent this overlap and the representation of domains across RBs. Of the 19 RBs included in the study, 42% (8/19) aimed to increase happiness or well-being; 42% (8/19) to facilitate mindfulness, meditation, or relaxation; 37% (7/19) to reduce negative emotions; and 37% (7/19) to support physical health (the purpose and theoretical orientation for each RB is provided in Multimedia Appendix 5). Less commonly, RBs aimed to support anxiety or stress (3/19, 16%), behavior change (2/19, 10%), relationships (2/19, 10%), alcohol or substance use (1/19, 5%), resilience (1/19, 5%), or sleep (1/19, 5%).

In terms of theoretical background or strategies, 58% of RBs (11/19) used advice or tips or strategies or skills training, 58% (11/19) used information or education, and 42% (8/19) used mindfulness or meditation techniques. The remaining and less common theoretical background or strategies included relaxation techniques (5/19, 26%), monitoring or tracking (4/19, 21%), goal setting (3/19, 16%), assessment (2/19, 10%), gratitude (2/19, 10%), CBT (behavioral) skills (1/19, 5%), or CBT (cognitive) skills (1/19, 5%). The A-MARS review of the evidence base for the RBs revealed that 18 (95%) have not been trialed or tested. One RB (Pain eHealth for Activity, Skills, and Education) has been trialed and outcome tested in 1 to 2 RCTs and reported positive results, but as this program requires access to be granted, our review was based on the “resources” tab of their website and not the program itself.

##### Apps

Each app also had multiple areas of focus for both the purpose and theoretical orientation. The following section represents this overlap, and the domains emphasized across all apps. Of the 42 reviewed apps, 42% (18/42) focused on increasing happiness and well-being (the purpose and theoretical orientation for each app is provided in Multimedia Appendix 6). In addition, 36% (15/42) aimed to support mindfulness, meditation, or relaxation, 31% (13/42) focused on reducing negative emotions, 21% (9/42) aimed to support behavior change, 21% (9/42) focused on reducing anxiety or stress, 17% (7/42) targeted relationship support, and 17% (7/42) aimed to improve physical health. Fewer programs targeted alcohol or substance use (4/42, 9%), PTSD (5/42, 12%), anger (4/42, 9%), sleep (2/42, 5%), depression (4/42, 9%), and goal setting (1/42, 2%).

In terms of the 42 apps’ theoretical background or strategies used, 62% (26/42) used advice, tips, strategies, or skills training, 60% (25/42) used information or education, 43% (18/42) used monitoring or tracking, 26% (11/42) used relaxation strategies, 24% (10/42) used mindfulness or meditation, and 24% (10/42) used goal setting. Less common theoretical background or strategies used included the following: acceptance and commitment therapy (1/42, 2%), assessment (6/42, 1%), CBT (cognitive) skills (7/42, 2%), CBT (behavioral) skills (7/42, 2%), cognitive processing therapy (CPT; 1/42, 2%), feedback (3/42, 7%), gratitude (1/42, 2%), prolonged exposure (PE; 1/42, 2%), resilience (1/42, 2%), skills training (1/42, 2%), and family resilience training (1/42, 2%). On the basis of the A-MARS evidence base review, the efficacy and effectiveness of apps are still in its infancy. In total, 33% (14/42) apps have not been trialed or tested. A total of 45% (19/42) of apps have been trialed by studies that are not RCTs with partially positive outcomes, or little or no contradictory evidence. Around 14% (6/42) of apps have been trialed by studies that are not RCTs, with positive outcomes and no contradictory evidence. Finally, 7% (3/42) of apps have been trialed and outcome tested in 1 to 2 RCTs with positive results reported.

### Step 3: Evaluation of DMHIs

#### Step 3: Procedures for Quality Review

Each RB and WBP was then evaluated via the A-MARS, and each app via the ARIA. A summary of key themes for each survey is described in the subsequent sections.

#### A-MARS: Quality Review of WBPs and RBs

##### Overview

All mean totals reported (ie, subscale mean, quality mean, and total mean scores) are based on the average ratings provided by 2 researchers (RRA and MAM) with the highest possible mean score being 5.0 (the mean scores for all domains for each e-tool are provided in Multimedia Appendix 7).

##### A-MARS Engagement

A higher average rating on the engagement subscale indicates greater levels of engagement, interest, customization, interactivity or interoperability, and target group fit. The average engagement mean rating was 2.93 (SD 0.52), ranging from 2.10 to 4.47. In total, 4 e-tools received an average score for engagement 1 SD above the mean—2 RBs (VA National Center for PTSD and VA Public Health) and 2 WBPs (AIMS for Anger Management and Misadventures in Money Management).

##### A-MARS Functionality

A higher score on the functionality subscale indicates high performance, ease of use, navigation, and design. This subscale received the highest overall ratings across all subscales with an average score 4.03 (SD 0.52), ranging from 3.00 to 4.88. The highest scoring e-tools (1 SD above the mean) for functionality were 3 WBPs (AIMS for Anger Management, Shield of Resilience Training, and Tactical Breather).

##### A-MARS Aesthetic

The aesthetic subscale represents the layout, graphics, and visual appeal of the e-tool, with a higher mean score representing a higher aesthetic rating. The average aesthetic mean rating was 3.62 (SD 0.40), ranging from 3.00 to 4.33. For aesthetics, 3 e-tools received an average score 1 SD above the mean—1 RB (About Face), and 2 WBPs (AIMS for Anger Management and HeadFIT).

##### A-MARS Information

The information subscale evaluates whether the e-tool contains high quality information (eg, text, feedback, measures, and references) from a credible source. A higher score for the information subscale indicates greater quality of information, greater quantity of information, precise goals, clear visual information, high credibility of sources, and strong evidence base. The average information subscale score was 3.54 (SD 0.73) and ranged from 1.67 to 4.80. The highest scoring e-tools (1 SD above the mean) for information were 3 RBs (Manage Stress: VA National Center for Health Promotion and Disease Prevention, National Sleep Foundation, and VA National Center for PTSD).

##### A-MARS Quality

The A-MARS quality subscale consists of the average rating across the engagement, functionality, aesthetic, and information subscales. Therefore, a higher score indicates higher overall quality without raters’ subjective quality and health-related information ratings. Average quality scores ranged from 2.51 to 4.19, with an average rating of 3.53 (SD 0.44). In terms of e-tool quality, 3 e-tools received an average rating 1 SD above the mean, 2 were WBPs (AIMS for Anger Management; Misadventures in Money Management), and one was an RB (Manage Stress: VA National Center for Health Promotion and Disease Prevention).

##### A-MARS Subjective Quality

Subjective quality subscale score was determined by assessing whether the researchers would recommend the tool, how many times they thought they would use the tool, whether they would pay for the tool, and their overall rating of the tool. A higher score represents a higher subjective quality rating. This subscale received the overall lowest ratings, with an average of 2.53 (SD 0.60), ranging from 1.75 to 4.25. There were 2 WBPs that scored 1 SD above the mean for the subjective quality domain (AIMS for Anger Management and Misadventures in Money Management).

##### A-MARS Health-Related Quality

Finally, the health-related quality subscale was assessed by rating the e-tools on subject matter related to whether there were additional resources provided, other strategies recommended for the user, multiple solutions offered for the presenting issue, multiple symptoms addressed, opportunities for real time tracking, and obvious access to health-related help. A higher score indicates greater health-related quality. This subscale had an average rating of 3.24 (SD 0.98) and ranged from 1.58 to 4.67. Within this domain, 1 WBP (AIMS for Anger Management) and 4 RBs (Manage Stress: VA National Center for Health Promotion and Disease Prevention, NHS Every Mind Matters, VA Make the Connection, and VA National Center for PTSD) received an average score 1 SD above the mean.

##### A-MARS Total

The A-MARS total mean score is a reflection of the overall quality of the RB and WBP (ie, e-tool), with an average score across all e-tools of 3.32 (SD 0.49), ranging from 2.38 to 4.11. There were 5 e-tools that scored 1 SD above the mean, 2 WBPs (AIMS for Anger Management and Misadventures in Money Management), and 3 RBs (Manage Stress: VA National Center for Health Promotion and Disease Prevention, VA Make the Connection, and VA National Center for PTSD).

#### ARIA: Quality Review of Apps

##### ARIA Part A

Part A was completed before downloading the app and assessed based on the app store page to evaluate goal fit, trustworthiness, privacy, and affordability. The average rating between the 2 researchers was 24.39 (SD 1.85) and ranged from a total score of 21 to 29 (the mean scores for each section of the ARIA for each app are provided Multimedia Appendix 8). In total, 7 apps received an average score 1 SD above the mean (Couples Coach, CPT Coach, Meditation Rx, OSI Connect, PeerConnect, PE Coach 2, and SwapMyMood).

In terms of individual item analysis, the researchers felt that the app fits the user’s (ie, military member, PSP, and veteran) purpose, with the average rating of 3.9 (SD 0.34), ranging from 3 (*neutral*) to 5 (*strongly agree*). The largest range was 2.0 (disagree) to 5.0 (strongly agree) on an item assessing whether the user can trust that relevant experts in the field developed the app, with an average rating of 3.6. Most apps on the app store page did not include a statement about the risks associated with using the app, with an average rating of 2.5 (SD 0.75), a range of 2 to 4.50, and 27 (64%) receiving a score of 2 (disagree). In terms of declaring a conflict of interest, only 1 app (*Mindarma*) provided a conflict of interest statement. Most apps (36/42, 86%) had a privacy policy that explained what information is collected by the app, who will have access to this information, and how the information will be used.

##### ARIA Part B

Part B was completed after using each app for a minimum of 10 minutes, using all links, and checking all sounds, videos, and tools associated with the app. A higher score on part B indicates a higher quality app related to trustworthiness, security, ease of use, functionality, usefulness, and satisfaction. Overall, the average rating was 43.38 (SD 3.92) and ranged from 36 to 50. In total, 6 apps scored 1 SD above the mean (Couples Coach, PTSD Coach, PTSD Family Coach, R2MR, VetChange, and Virtual Hope Box).

##### ARIA Subjective Quality

The subjective quality of each app was assessed via 2 items. First, 2 researchers rated whether they would recommend the app, with an average rating of 3.73 (SD 0.52), ranging from 2.5 to 5. There were 5 apps that received an average score 1 SD above the mean (AIMS for Anger Management, FOCUS on the Go!, Insomnia Coach, PTSD Family Coach, and R2MR).

The researchers also rated the number of stars that they felt represented the overall quality of the app. The average rating for this item was 3.08 (SD 0.69) and ranged from 1.5 to 4.5. There were 8 apps that scored 1 SD above the mean (AIMS for Anger Management, CBT-Insomnia Coach, Couples Coach, COVID Coach, FOCUS on the Go!, Mindfulness Coach, R2MR, and Virtual Hope Box).

## Discussion

### Principal Findings

This environmental scan was conducted to review, describe, and qualitatively evaluate DMHIs recommended for military member, PSP, and veteran populations. This information is vital as high quality, accessible care for military members, PSP, and veterans is sorely needed [38]. This environmental scan reviewed 42 mobile apps, 19 RBs, and 8 WBPs, totaling to 69 DMHIs.

According to our review, 55% (38/69) of the apps, RBs, and WBPs have not been trialed or tested, while 30% (21/69) of the apps and WBPs have been trialed by studies that are not RCTs (eg, acceptability, usability, and satisfaction ratings) with partially positive outcomes or little to no contradictory evidence. In total, 10% (7/69) of the apps and WBPs have been trialed by studies that are not RCTs (eg, acceptability, usability, and satisfaction ratings) and have positive outcomes with no contradictory evidence. Finally, 6% (4/69), 3 apps (Drinks:Ration, Virtual Hope Box, and VetChange), and 1 RB (Pain eHealth for Activity, Skills, and Education), have been trialed and outcome tested in 1 to 2 RCTs and recorded positive results. Although Pain eHealth for Activity, Skills, and Education is a WBP that has been trialed and tested, but as this program requires access to be granted to the user, our review was based on the “resources” tab of their website, and not the program itself.

### DMHI Summary

#### Summary Based on Aim, Strategy, Quality, and Evidence

Across apps, RBs, and WBPs, the most common aim was to increase happiness and well-being, and the most common strategies used were advice, tips, strategies, and skills training. In terms of the highest rated DMHIs, AIMS for Anger Management received the highest quality mean score and the highest total mean score (A-MARS), and PTSD Family Coach was rated the highest for overall quality (ARIA part B). SwapMyMood received the highest rating for overall fit, trustworthiness, privacy, and affordability (ARIA part A). In total, 2 e-tools received the highest score on 2 A-MARS subscales: Manage Stress: VA National Center for Health Promotion and Disease Prevention (information and health-related quality) and Misadventures in Money Management (engagement and subjective quality). No app, RB, or WBP has been trialed and outcome tested in more than 3 high quality RCTs with positive results.

#### Summary Based on Population

#### Summary Section Organization

The following section will be used to discuss the quality and usability of the highly rated DMHIs (ie, DMHIs that received a rating 1 SD above the mean) on the A-MARS [33] and the ARIA [35] and organized by the 3 populations of interest. Each of these highly rated DMHIs will be organized and synthesized based on the A-MARS levels of evidence [33], and the Canadian Psychological Association Task Force’s [39] recommendations for evidence-based practice (EBP) will be used as a framework to interpret these results.

#### Military Members

In total, 2 apps received a high rating on ARIA subscales with promising initial results, including PTSD Coach, which has been trialed for military member or veteran populations in pilot RCT and non-RCT satisfaction and useability studies, and Virtual Hope Box, which has been evaluated in proof-of-concept and RCT studies. PTSD Coach (also for veteran populations) is an app developed to support individuals with PTSD through psychoeducation and self-management tools and received a high rating on ARIA part B, which indicates high levels of trustworthiness, ease of use, usefulness, and user satisfaction. Virtual Hope Box, designed to support stress management and emotion regulation, has promising evidence and also scored highly on the ARIA part B. These apps are recommended for military members as they have been trialed through initial studies and received high ratings related to the overall quality of the apps; however, multiple efficacy trials have not been conducted to date.

One app, Drinks:Ration (also for veteran populations), was also trialed with 1 or 2 RCT studies, with positive initial results. Although this is the case, this app did not receive a high rating (1 SD above the mean) on any of the ARIA subscales, suggesting that this app is likely efficacious at supporting individuals in managing and monitoring alcohol consumption, but, in terms of user satisfaction and app quality (as assessed by the ARIA), this app comparatively did not score in the upper percentile. In addition, the ARIA assesses constructs related to app security, privacy, and trustworthiness, which may not impact the overall app efficacy. Therefore, this app is recommended to military members as a helpful app for monitoring and potentially reducing their drinking, and future research is suggested to improve app security, privacy, functionality, and trustworthiness.

Next, there were a number of DMHIs recommended for military members that have trailed initially (eg, acceptability studies), have partially positive outcomes (in non-RCT studies), and have little or no contrary evidence. AIMS for Anger Management (also for veteran populations) is a WBP developed to provide individuals with tools to track and manage anger, with information about anger, and with opportunities for support. AIMS for Anger Management received high scores across many domains, suggesting that this WBP is highly engaging, interesting, aesthetically pleasing, functional, and high quality. Next, HeadFIT, a WBP developed to support individuals with stress management skills, received a high rating on the aesthetic subscale indicating that it is aesthetically pleasing; however, it did not rank highly in other areas related to quality or information. PE Coach 2, an app with the goal of providing psychoeducation about PTSD-related symptoms and coping tools, scored highly on the ARIA part A, suggesting that it is trustworthy, has adequate privacy, and is affordable. PE Coach was assessed via RCT; however, the intervention condition included multiple apps and therefore it is difficult to discern the efficacy of PE Coach on its own. Next, PTSD Family Coach (also for veteran populations and their families) was developed to support family members of those living with PTSD, and has been assessed with a pilot RCT, feasibility, and acceptability study. This app received a high rating on ARIA part B indicating that the app is functional, useful, and satisfactory. Finally, R2MR (also for PSP and veteran populations) was rated highly for ARIA part B. This app was developed, as an adjunct to in-person R2MR training, to support short-term performance and long-term mental health outcomes. These apps are recommended as potentially helpful. Importantly, a stance of EBP must continue to be maintained, and further research is needed on each of these DMHIs to suggest them as effective tools for military members [39].

Finally, of the DMHIs rated highly on either the ARIA or A-MARS subscales, there were 4 that have not been trialed and tested. First, Misadventures in Money Management received high ratings related to engagement and overall quality. Second, NHS Every Mind Matters was rated positively for health-related information. Third, Tactical Breather received a high rating related to its ease of use. Importantly, Tactical Breather has been assessed via RCT, however, the intervention condition included the use of multiple other apps; therefore, it is difficult to discern and determine the efficacy of this app. Finally, SwapMyMood (also for veteran populations) was rated highly regarding its fit for the population, trustworthiness, privacy, and affordability. These DMHIs appear engaging, secure, and informative, and were rated highly in terms of user experience. While these are all important considerations, these DMHIs cannot be definitively recommended to military members until more evidence, in particular effectiveness and efficacy trials, support their use with this population [39].

#### Public Safety Personnel

In total there were 3 DMHIs that received high ratings on the ARIA or A-MARS subscales that are recommended for and trialed with PSP populations. PeerConnect was developed to provide mental health service to support wellness and belonging. This app has a high rating on the ARIA part A suggesting that it provides information on app security and privacy and appears to be a good fit for PSP. PeerConnect has promising preliminary evidence such that it has been trialed with PSP populations in non-RCT studies (eg, satisfaction and acceptability), with partially positive outcomes or little or no contradictory evidence. R2MR (also for military member and veteran populations), meant to support short-term health and long-term mental health, received high ratings on the ARIA part B (see Military Members section) and has similar positive preliminary evidence to PeerConnect. Finally, Shield of Resilience Training has not been trialed or tested for PSP but received a high rating on functionality suggesting that it is easy to use and navigate. Overall, there are few DMHIs recommended for or trialed with PSP populations that also received a high rating. There are no DMHIs for PSP that received a high rating and have a strong evidence base. As per the Canadian Psychological Association Task Force’s recommendations, more research is needed before specific DMHIs can be recommended for PSP [39], and this is a notable gap as PSP do not have many high quality DMHIs developed for them.

Notably, there were 3 apps (Insight Timer, Mindfulness Coach, and Mindshift) that were noted for higher levels of evidence (ie, have been trialed in non-RCT trials and have positive outcomes with no contradictory results) but did not receive high ratings on the ARIA. These apps were made for the public, and therefore, were only recommended as potential resources for PSP populations. In addition, these apps have not been studied specifically with PSP populations and instead have been recommended for these populations and trialed with the general population. These apps with promising evidence may have had lower ratings because the measures used to assess these apps generally evaluate app satisfaction, usability, and security or privacy, and do not assess app effectiveness.

#### Veterans

In total, 16 DMHIs received high ratings on either the A-MARS or ARIA for veteran populations. Around 2 apps received high ratings related to ARIA part B, indicating that they are secure, trustworthy, usable, and functional, and have also been trialed via pilot RCT, RCT, and non-RCT (eg, usability, satisfaction, effectiveness trials) studies, with positive initial outcomes. These apps include, first, PTSD Coach (also for military members, assessed via pilot RCT), which supports individuals in managing PTSD symptoms, and second, VetChange, an app developed to reduce drinking and to support PTSD symptoms (evaluated by one RCT with military members indicating positive results). These apps are recommended for veteran populations based on preliminary research with positive results and based on high ratings related to user experience. Again, although they have positive research outcomes reported in preliminary RCT studies, in line with EBP, additional studies to assess internal and external validity are necessary to effectively address the empirical evidence supporting these programs [39].

In total, 7 DMHIs scored highly on subscales related to the ARIA and A-MARS and have also been trialed via non-RCT studies (eg, acceptability) with partially positive outcomes or little to no contradictory evidence: (1) AIMS for Anger Management (also for military members) was developed to provide users with tools to track and manage their anger, with education about anger, and with opportunities to find support. This WBP received high ratings on A-MARS engagement, functionality, aesthetic, quality, subjective quality, and health-related quality, suggesting that it is of high quality; (2) Couples Coach, an app designed to support partners in improving their relationship and exploring new ways for connection, scored high on the ARIA part A and part B. This suggests that it is secure, private, affordable, usable, and functional; (3) CPT Coach is an app developed to support individuals participating in CPT by providing worksheets, readings, and symptom monitoring tools. This app received a high score on ARIA part A, suggesting it is affordable, secure, and private; (4) Meditation Rx, an app designed to support stress reduction through guided meditations, also received a high score on ARIA part A (eg, security and privacy); (5) OSI Connect, an app with the aim of teaching self-management tools to assist with common mental health concerns, also received a high score on ARIA part A (eg, security and privacy); (6) PTSD Family Coach (also for military members) aims to support family members of those with PTSD (evaluated by pilot RCT, feasibility, and acceptability trial). This app received a high score on ARIA part B, suggesting it is secure, functional, and user friendly; (7) R2MR (also for military members and PSPs), an app developed to support (adjunct to in-person training) short-term performance and long-term mental health received a high score on ARIA part B, suggesting that it is also secure, functional, and user friendly. Overall, these DMHIs for veteran populations have preliminary evidence supporting their use, with partially positive outcomes or little to no contradictory evidence. On the basis of our review of the user experience, these DMHIs received the highest rating and may be used by veteran populations. However, in line with EBP, these DMHIs require further research, including effectiveness and efficacy trials [39].

In total, 7 DMHIs recommended for veteran populations do not appear to have evidence supporting their use and have not been trialed or tested: (1) AboutFace received high ratings by the researchers on aesthetics; (2) Manage Stress: VA National Center for Health Promotion and Disease Prevention received high ratings across A-MARS subscales (information, quality, health-related quality, and total score); (3) National Sleep Foundation scored highly on information; (4) SwapMyMood (also for military members) received a high score related to the ARIA part A, which indicates that it is affordable, secure, and private; (5) VA Make the Connection scored highly on health-related quality and total score; (6) VA National Center for PTSD scored highly across many A-MARS subscales, including engagement, information, health-related quality, and total score; and (7) VA Public Health scored highly on the engagement subscale. Although these DMHIs received high quality ratings based on their usability, experience, and information, in line with EBP [39], these must be trialed and tested before they are recommended for veteran populations.

### Limitations and Strengths of the Environment Scan

Some methodological limitations exist in this environmental scan. Only DMHIs that were in English and available in Canada were included, which may limit the scope of this review. A further potential limitation is that there was no clear definition for RBs and WBPs in literature. Instead, the research team engaged in discussions to differentiate RBs and WBPs from other web-based programs (eg, web-based courses, chat rooms, Facebook groups, and YouTube channels). The research team decided a web-based program was an *interactive web-based platform* that does not involve an invitation or log-in code, and an RB was a web-based platform that was not interactive but provided *key information* or *links to the user*. In the future, it would be beneficial to have a clear definition and to differentiate between the aforementioned web-based resources.

In addition, many of the DMHIs have not been formally evaluated to determine their fit with the target populations, or to determine the efficacy or effectiveness at supporting users’ well-being or resilience. Therefore, these are subjective ratings and assessments of fit and quality and do not represent the effectiveness or efficacy. The research team that completed this review has worked closely with military members, PSP, and veteran populations; however, they are not members of these populations. This limitation may have impacted the subjective quality rating and fit for population rating, as it was the researchers’ perspectives and not target population members’ perspectives.

There are also several strengths related to this environmental scan. An extensive search of 6 databases, gray literature (ie, Google search), and 12 targeted websites were completed, strengthening the scope of this review. We also established clear inclusion and exclusion criteria that were refined throughout. This project also applied a whole-person and multidimensional well-being approach to evaluate the programs. This framework prioritizes multiple dimensions of wellness to emphasize one’s well-being within and across domains, allowing researchers and clinicians to focus on protective networks rather than deficit-based frameworks. Finally, scales developed for the purpose of reviewing app and e-tool quality were used to organize and critically analyze the included DMHIs.

### Future Directions

Overall, our findings are consistent with Miralles et al [29], who suggested that, for mobile apps, research is lacking, that they are not empirically validated interventions, and that there are privacy and confidentiality concerns. There has been a surge of DMHIs because of the COVID-19 pandemic, and the current evidence base for these interventions reflects this novelty. It is imperative that researchers aim to continue to push for EBP, even for self-managed DMHIs. Broadly, the evidence base for DMHIs for military members, PSP, and veterans is weak, with over half of them not having been trialed or tested, and no program being assessed with 3 or more RCTs with positive results. Efficacy and effectiveness trials are key to progressing EBP of DMHIs, and therefore, must be prioritized [39].

The current literature is yet in the early stages, with many studies focusing on acceptability, usability, or initial pilot studies. An important next step in establishing the evidence for DMHI will be conducting carefully controlled RCTs to demonstrate the efficacy of their use. In addition, developers must continue to consider the population characteristics and treatment preferences of those they are creating the program for [39]. It is key that future DMHI developers and researchers maintain a multidimensional well-being approach to support military members, PSP, and veterans across well-being domains, and to support their resilience [16,17].

### Conclusions

This environmental scan contributes to the literature by collecting and reviewing available DMHIs intended to promote resilience and well-being in military members, PSP, and veterans. The purpose of this study was to determine the quality of DMHIs. Using an environmental scan methodology, we were able to capture the breadth and depth of literature and available resources (without research literature) to review and categorize the available DMHIs recommended or developed for these populations. Although the results of this environmental scan highlight key apps, WBPs, and RBs that are higher in quality, there is little research that supports their effectiveness and efficacy. Further research is needed before DMHIs can be confidently promoted and widely distributed.

## Appendix

Multimedia Appendix 1 Search terms and examples.

Multimedia Appendix 2 Google search terms.

Multimedia Appendix 3 Targeted website search.

Multimedia Appendix 4 Web-based program purpose and theoretical background.

Multimedia Appendix 5 Resource bank purpose and theoretical background.

Multimedia Appendix 6 App purpose and theoretical background.

Multimedia Appendix 7 Mean adapted Mobile App Rating Scale ratings.

Multimedia Appendix 8 Mean Alberta Rating Index for Apps ratings.

## Abbreviations

A-MARS: adapted Mobile App Rating Scale
ARIA: Alberta Rating Index for Apps
CBT: cognitive behavioral therapy
CPT: cognitive processing therapy
DMHI: digital mental health intervention
EBP: evidence-based practice
e-tool: electronic tool
ICC: intraclass correlation coefficient
MARS: Mobile App Rating Scale
PE: prolonged exposure
PPTE: potentially psychologically traumatic event
PRISMA: Preferred Reporting Items for Systematic Reviews and Meta-Analyses
PRISMA-ScR: Preferred Reporting Items for Systematic Reviews and Meta-Analyses Extension for Scoping Reviews
PSP: public safety personnel
PTSD: posttraumatic stress disorder
RB: resource bank
RCT: randomized controlled trial
WBP: web-based program
