# Supplementary material for: Digital Health Resilience and Well-Being Interventions for Military Members, Veterans, and Public Safety Personnel: Environmental Scan and Quality Review
Source: JMIR Mhealth Uhealth. 2025 Apr 1;13:e64098. doi: 10.2196/64098 (PMC12000787; doi:10.2196/64098)
Supplement: Multimedia Appendix 1 [file mhealth_v13i1e64098_app1.docx]

Example from Ovid Search

Concept 1:

(Public Safety Personnel OR police* OR firefighter* OR first responder* OR firem* OR military OR soldier* OR army OR special force* OR (active AND duty) OR paramilitary OR armed-force* OR armed-service* OR servicewom* OR servicem*n OR air-personnel OR defence-force* OR defense-force* OR service-personnel OR navy OR air-force OR infantryman OR civil defence OR civil defense OR medic OR enlisted personnel OR reserve personnel OR Royal Canadian Mounted Police OR RCMP OR officer* OR Emergency Medical Technician* OR EMT OR Emergency Medical Service* OR EMS OR paramedic* OR veteran* OR VA):ti, ab

Concept 2:

(resilien* OR coping OR hardiness OR grit* OR flourishing OR wellbeing OR well-being OR emotion regulation OR affect OR (self-manag* AND symptom*) OR mood): ti, ab

Concept 3:

(virtual game OR online game OR gaming OR virtual play OR online play OR videogam* OR smart phone OR mobile app* OR ((Internet based) AND (intervention OR program)) OR ((online) AND (intervention OR program)) OR ((web based) AND (intervention OR program)) OR mental health app* OR smartphone app* OR smartphone-based OR smartphone application OR computerized OR computer-based OR iPAD OR computer based OR Internet format): ti, ab

Limits: English articles from 2000 onwards

Search fields: Titles and Abstracts

Search Mode: Boolean
