# Supplementary material for: Digital Health Resilience and Well-Being Interventions for Military Members, Veterans, and Public Safety Personnel: Environmental Scan and Quality Review
Source: JMIR Mhealth Uhealth. 2025 Apr 1;13:e64098. doi: 10.2196/64098 (PMC12000787; doi:10.2196/64098)
Supplement: Multimedia Appendix 2 [file mhealth_v13i1e64098_app2.docx]

Google Search

| Search # | Search description | # of Hits |
| --- | --- | --- |
| 1 | Resilience virtual intervention  Public-safety-personnel OR police OR firefighter OR first-responder OR military OR paramedic OR veteran | 21,900,000 |
| 2 | Resilience online intervention  Public-safety-personnel police firefighter first-responder military paramedic veteran | 28,000,000 |
| 3 | Resilience digital intervention  Public-safety-personnel police firefighter first-responder military paramedic veteran | 23,300,000 |
| 4 | Resilience mobile app intervention  Public-safety-personnel police firefighter first-responder military paramedic veteran | 18,700,000 |
| 5 | Resilience e-health intervention  Public-safety-personnel police firefighter first-responder military paramedic veteran | 26,600,000 |
| 6 | Resilience m-health intervention  Public-safety-personnel police firefighter first-responder military paramedic veteran | 34,300,000 |
| 7 | Resilience virtual program  Public-safety-personnel police firefighter first-responder military paramedic veteran | 35,200,000 |
| 8 | Resilience online program  Public-safety-personnel police firefighter first-responder military paramedic veteran | 35,700,000 |
| 9 | Resilience mobileapp program  Public-safety-personnel police firefighter first-responder military paramedic veteran | 1,600,000 |
| 10 | Resilience digital program  Public-safety-personnel police firefighter first-responder military paramedic veteran | 60,600,000 |
| 11 | Resilience e-health program  Public-safety-personnel police firefighter first responder military paramedic veteran | 290,000,000 |
| 12 | Resilience m-health program  Public safety personnel police firefighter first responder military paramedic veteran | 345,000,000 |
| 13 | Resilience virtual game  Public safety personnel police firefighter first responder military paramedic veteran | 76,400,000 |
| 14 | Resilience online game  Public safety personnel police firefighter first responder military paramedic veteran | 121,000,000 |
| 15 | Resilience mobileapp game  Public safety personnel police firefighter first responder military paramedic veteran | 4,160,000 |
| 16 | Resilience e-health game  Public safety personnel police firefighter first responder military paramedic veteran | 84,400,000 |
| 17 | Resilience m-health game  Public safety personnel police firefighter first responder military paramedic veteran | 71,500,000 |
| 18 | Resilience digital game  Public safety personnel police firefighter first responder military paramedic veteran | 143,000,000 |
| 19 | Wellbeing virtual intervention  Public-safety-personnel police firefighter first-responder military paramedic veteran | 11,500,000 |
| 20 | Wellbeing online intervention  Public-safety-personnel police firefighter first-responder military paramedic veteran | 40,200,000 |
| 21 | Wellbeing digital intervention  Public-safety-personnel police firefighter first-responder military paramedic veteran | 19,500,000 |
| 22 | Wellbeing mobileapp intervention  Public-safety-personnel police firefighter first-responder military paramedic veteran | 859,000 |
| 23 | Wellbeing e-health intervention  Public-safety-personnel police firefighter first-responder military paramedic veteran | 58,000,000 |
| 24 | Wellbeing m-health intervention  Public-safety-personnel police firefighter first-responder military paramedic veteran | 72,600,000 |
| 25 | Wellbeing virtual program  Public-safety-personnel police firefighter first-responder military paramedic veteran | 20,200,000 |
| 26 | Wellbeing online program  Public-safety-personnel police firefighter first-responder military paramedic veteran | 23,800,000 |
| 27 | Wellbeing mobileapp program  Public-safety-personnel police firefighter first-responder military paramedic veteran | 2,890,000 |
| 28 | Wellbeing digital program  Public-safety-personnel police firefighter first-responder military paramedic veteran | 87,700,000 |
| 29 | Wellbeing e-health program  Public-safety-personnel police firefighter first-responder military paramedic veteran | 298,000,000 |
| 30 | Wellbeing m-health program  Public-safety-personnel police firefighter first-responder military paramedic veteran | 302,000,000 |
| 31 | Wellbeing virtual game  Public-safety-personnel police firefighter first-responder military paramedic veteran | 16,500,000 |
| 32 | Wellbeing online game  Public-safety-personnel police firefighter first-responder military paramedic veteran | 68,200,000 |
| 33 | Wellbeing mobileapp game  Public-safety-personnel police firefighter first-responder military paramedic veteran | 1,800,000 |
| 34 | Wellbeing e-health game  Public-safety-personnel police firefighter first-responder military paramedic veteran | 71,200,000 |
| 35 | Wellbeing m-health game  Public-safety-personnel police firefighter first-responder military paramedic veteran | 56,500,000 |
| 36 | Wellbeing digital game  Public-safety-personnel police firefighter first-responder military paramedic veteran | 29,000,000 |
| 37 | Well-being virtual intervention  Public-safety-personnel police firefighter first-responder military paramedic veteran | 142,000,000 |
| 38 | Well-being online intervention  Public-safety-personnel police firefighter first-responder military paramedic veteran | 158,000,000 |
| 39 | Well-being digital intervention  Public-safety-personnel police firefighter first-responder military paramedic veteran | 67,100,000 |
| 40 | Well-being mobileapp intervention  Public-safety-personnel police firefighter first-responder military paramedic veteran | 81,800,000 |
| 41 | Well-being e-health intervention  Public-safety-personnel police firefighter first-responder military paramedic veteran | 160,000,000 |
| 42 | Well-being m-health intervention  Public-safety-personnel police firefighter first-responder military paramedic veteran | 145,000,000 |
| 43 | Well-being virtual program  Public-safety-personnel police firefighter first-responder military paramedic veteran | 1,680,000,000 |
| 44 | Well-being online program  Public-safety-personnel police firefighter first-responder military paramedic veteran | 1,610,000,000 |
| 45 | Well-being mobileapp program  Public-safety-personnel police firefighter first-responder military paramedic veteran | 1,210,000,000 |
| 46 | Well-being digital program  Public-safety-personnel police firefighter first-responder military paramedic veteran | 881,000,000 |
| 47 | Well-being e-health program  Public-safety-personnel police firefighter first-responder military paramedic veteran | 1,300,000,000 |
| 48 | Well-being m-health program  Public-safety-personnel police firefighter first-responder military paramedic veteran | 1,270,000,000 |
| 49 | Well-being virtual game  Public-safety-personnel police firefighter first-responder military paramedic veteran | 456,000,000 |
| 50 | Well-being online game  Public-safety-personnel police firefighter first-responder military paramedic veteran | 1,760,000,000 |
| 51 | Well-being mobileapp game  Public-safety-personnel police firefighter first-responder military paramedic veteran | 598,000,000 |
| 52 | Well-being e-health game  Public-safety-personnel police firefighter first-responder military paramedic veteran | 278,000,000 |
| 53 | Well-being m-health game  Public-safety-personnel police firefighter first-responder military paramedic veteran | 233,000,000 |
| 54 | Well-being digital game  Public-safety-personnel police firefighter first-responder military paramedic veteran | 182,000,000 |
