# Supplementary material for: Digital Health Resilience and Well-Being Interventions for Military Members, Veterans, and Public Safety Personnel: Environmental Scan and Quality Review
Source: JMIR Mhealth Uhealth. 2025 Apr 1;13:e64098. doi: 10.2196/64098 (PMC12000787; doi:10.2196/64098)
Supplement: Multimedia Appendix 3 [file mhealth_v13i1e64098_app3.docx]

Targeted website search

| **Website** | **Hyperlink** |
| --- | --- |
| Australia Department of Defence | https://www.defence.gov.au/ |
| Canada Department of Defence | https://www.canada.ca/en/department-national-defence.html |
| Canadian Armed Forces | https://forces.ca/en/?utm_campaign=dnd-mdn-sem-22-23&utm_medium=sem&utm_source=ggl&utm_content=ad-text-en&utm_term=canadian%20armed%20forces&adv=2324-409731&id_campaign=19904845882&id_source=147148894509&id_content=652798016570&gclid=Cj0KCQjwoeemBhCfARIsADR2QCuIT_gwEemqNtM1hQUNaVx84Z_O_XsPyS0k4fv28sA-ofvTJ9j__rIaAnaGEALw_wcB&gclsrc=aw.ds |
| Canadian Institute for Public Safety Research and Treatment | https://www.cipsrt-icrtsp.ca/ |
| Edmonton Fire | https://www.edmonton.ca/programs_services/about-fire-rescue-services |
| Edmonton Police | https://www.edmontonpolice.ca/ |
| New Zealand Ministry of Defence | https://www.defence.govt.nz/ |
| Royal Canadian Mounted Police | https://www.rcmp-grc.gc.ca/ |
| United Kingdom Ministry of Defence | https://www.gov.uk/government/organisations/ministry-of-defence |
| United States of America Department of Defense | https://www.defense.gov/ |
| Veterans Affairs | https://www.va.gov/ |
| Veterans Affairs Canada | https://www.veterans.gc.ca/eng |
