# Supplementary material for: Digital Health Resilience and Well-Being Interventions for Military Members, Veterans, and Public Safety Personnel: Environmental Scan and Quality Review
Source: JMIR Mhealth Uhealth. 2025 Apr 1;13:e64098. doi: 10.2196/64098 (PMC12000787; doi:10.2196/64098)
Supplement: Multimedia Appendix 4 [file mhealth_v13i1e64098_app4.docx]

WBPs purpose and theoretical background

| **Name of Web-Based Program** | **Focus (purpose): What the app targets** | **Theoretical background/Strategies** |
| --- | --- | --- |
| AIMS for Anger Management | - Increase happiness or well-being - Mindfulness, meditation, or relaxation - Reduce negative emotions - Anger | - Information or education - Goal setting - Advice, tips, strategies, or skills training - CBT (behavioural) - CBT (cognitive) |
| Freedom Qi Gong | - Increase happiness or well-being - Mindfulness, meditation, or relaxation - Anxiety or stress - Physical health | - Advice, tips, strategies, or skills training - Mindfulness or meditation - Relaxation - Gratitude |
| HeadFIT | - Increase happiness or well-being - Reduce negative emotions - Anxiety or stress | - Goal setting - Advice, tips, strategies, or skills training - CBT (behavioural) - CBT (cognitive) - Mindfulness or meditation - Relaxation - Strengths based |
| Misadventures in Money Management | - Behaviour change - Goal setting - Other (financial management) | - Advice, tips, strategies, or skills training |
| Provider Resilience | - Increase happiness or well-being - Mindfulness, meditation, or relaxation - Other (support resilience) | - Information or education - Monitoring or tracking - Mindfulness or meditation |
| Shield of Resilience Training | - Increase happiness or well-being - Reduce negative emotions - Behaviour change - Relationships | - Information or education - Advice, tips, strategies, or skills training |
| Tactical Breather | - Mindfulness, meditation, or relaxation - Anxiety or stress | - Relaxation |
| Tao Connect | - Increase happiness or well-being | - Information or education - Monitoring or tracking - CBT (behavioural) - CBT (cognitive) - Mindfulness or meditation - Gratitude |
