# Supplementary material for: Digital Health Resilience and Well-Being Interventions for Military Members, Veterans, and Public Safety Personnel: Environmental Scan and Quality Review
Source: JMIR Mhealth Uhealth. 2025 Apr 1;13:e64098. doi: 10.2196/64098 (PMC12000787; doi:10.2196/64098)
Supplement: Multimedia Appendix 5 [file mhealth_v13i1e64098_app5.docx]

RBs purpose and theoretical background

| **Resource Bank** | **Focus (purpose)** | **Theoretical background/Strategies** |
| --- | --- | --- |
| AboutFace | - Increase happiness or well-being - Reduce negative emotions - Physical health | - Information or education - Advice, tips, strategies, or skills training |
| Chris Germer Meditations | - Mindfulness, meditation, or relaxation | - Mindfulness or meditation - Relaxation - Gratitude |
| Exalted Warrior Foundation | - Mindfulness, meditation, or relaxation | - Mindfulness or meditation - Relaxation - Gratitude |
| First Responders First | - Mindfulness, meditation, or relaxation | - Assessment - Information or education - Monitoring or tracking - Advice, tips, strategies, or skills training |
| Head to Health | - Increase happiness or well-being - Mindfulness, meditation, or relaxation | - Information or education - Advice, tips, strategies, or skills training |
| Manage Stress: VA National Center for Health Promotion and Disease Prevention | - Increase happiness or well-being - Mindfulness, meditation, or relaxation - Anxiety or stress - Physical health | - Information or education - Advice, tips, strategies, or skills training |
| Meditation Oasis Podcasts | - Mindfulness, meditation, or relaxation | - Mindfulness or meditation - Relaxation |
| Mind Resilience Intervention | - Reduce negative emotions - Anxiety or stress - Relationships - Physical health - Other (resilience) | - Information or education - Advice, tips, strategies, or skills training |
| National Sleep Foundation | - Behaviour change - Other (sleep) | - Information or education - Monitoring or tracking - Goal setting - Advice, tips, strategies, or skills training |
| NHS Every Mind Matters | - Increase happiness or well-being - Reduce negative emotions | - CBT (behavioural) - Mindfulness or meditation |
| Pain and Opioid Safety | - Behaviour change - Alcohol or substance Use - Physical health | - Information or education - Goal setting - Advice, tips, strategies, or skills training |
| Pain eHealth for Activity, Skills, and Education (Resources) | - Reduce negative emotions - Physical health | - Advice, tips, strategies, or skills training - CBT (cognitive) |
| Responder Strong | - Increase happiness or well-being | - Assessment - Information or education - Goal setting - Mindfulness or meditation |
| Ten Percent Happier | - Anxiety or stress | - Mindfulness or meditation - Relaxation |
| VA Make the Connection | - Increase happiness or well-being - Reduce negative emotions - Relationships | - Information or education - Monitoring or tracking - Advice, tips, strategies, or skills training |
| VA National Center for PTSD | - Reduce negative emotions | - Information or education - Monitoring or tracking - Advice, tips, strategies, or skills training |
| VA Public Health | - Increase happiness or well-being - Reduce negative emotions - Physical health | - Information or education - Advice, tips, strategies, or skills training |
| Veterans Yoga Project | - Mindfulness, meditation, or relaxation - Physical health | - Mindfulness or meditation - Relaxation |
| Yoga Journal | - Increase happiness or well-being - Mindfulness, meditation, or relaxation | - Mindfulness or meditation |
