# Supplementary material for: Digital Health Resilience and Well-Being Interventions for Military Members, Veterans, and Public Safety Personnel: Environmental Scan and Quality Review
Source: JMIR Mhealth Uhealth. 2025 Apr 1;13:e64098. doi: 10.2196/64098 (PMC12000787; doi:10.2196/64098)
Supplement: Multimedia Appendix 6 [file mhealth_v13i1e64098_app6.docx]

Apps purpose and theoretical background

| **Name of App** | **Focus (purpose): What the app targets** | **Theoretical background/Strategies** |
| --- | --- | --- |
| 7-Minute Chi | - Mindfulness, meditation, or relaxation - Physical health | - Information or education - Advice, tips, strategies, or skills training - Mindfulness or meditation - Relaxation |
| ACT Coach | - Increase happiness or well-being - Mindfulness, meditation, or relaxation | - Information/Education - Monitoring or tracking - ACT |
| AIMS for Anger Management | - Increase happiness or well-being - Mindfulness, meditation, or relaxation - Reduce negative emotions - Anger | - Information or education - Goal setting - Advice, tips, strategies, or skills training - CBT (behavioural) - CBT (cognitive) |
| Beyond MST | - Increase happiness or well-being - Mindfulness, meditation, or relaxation - Reduce negative emotions - Depression - Anxiety or stress - Anger | - Assessment - Information or education - Monitoring or tracking - Goal setting - Advice, tips, strategies, or skills training - CBT (behavioural) - CBT (cognitive) |
| Breathe2Relax | - Mindfulness, meditation, or relaxation - Reduce negative emotions - Anxiety or stress - Anger | - Feedback - Relaxation |
| Calm | - Mindfulness, meditation, or relaxation | - Mindfulness or meditation - Relaxation |
| CBT-insomnia Coach | - Behaviour change - Other (sleep) | - CBT (behavioural) - CBT (cognitive_ |
| Chills Drill | - Mindfulness, meditation, or relaxation - Reduce negative emotions | - Advice, tips, strategies, or skills training - Mindfulness or meditation - Relaxation |
| Comfort Talk Pro App | - Mindfulness, meditation, or relaxation | - Relaxation |
| Couples Coach | - Relationships | - Assessment - Advice, tips, strategies, or skills training |
| COVID Coach | - Increase happiness or well-being - Reduce negative emotions - Relationships | - Monitoring or tracking - Advice, tips, strategies, or skills training |
| CPT Coach | - Reduce negative emotion | - Monitoring or tracking - Other (cognitive processing) |
| CrewCare | - Increase happiness or well-being - Anxiety or stress | - Information or education |
| Daily Yoga | - Increase happiness or well-being - Mindfulness, meditation, or relaxation - Physical health | - Advice, tips, strategies, or skills training - Mindfulness or meditation - Relaxation - Gratitude |
| Drinks:Ration | - Behaviour change - Alcohol or substance ese - Goal setting - Physical health | - Information or education - Monitoring or tracking - Goal Setting |
| Driven Resilience App | - Increase happiness or well-being - Mindfulness, meditation, or relaxation - Reduce negative emotions - Depression - Anxiety or stress - Relationships | - Assessment - Information or education - Monitoring or tracking - Advice, tips, strategies, or skills training - CBT (behavioural) - CBT (cognitive) - Other (resilience) |
| equipt | - Increase happiness or well-being - Reduce negative emotions - Anxiety or stress - Relationships - Physical Health | - Information or education - Monitoring or Tracking - Advice, tips, strategies, or skills training |
| eQuoo | - Increase happiness or well-being | - Information or education - Monitoring or tracking - Goal Setting - Advice, tips, strategies, or skills training |
| FOCUS on the Go! | - Increase happiness or well-being - Relationships | - Information or education - Advice, tips, strategies, or skills training - Other (family resilience training) |
| Insight Timer | - Mindfulness, meditation, or relaxation - Anxiety or stress - Behaviour change | - Mindfulness or meditation - Relaxation |
| Insomnia Coach | - Behaviour change - Other (sleep) | - CBT (behavioural) - CBT (cognitive) |
| Lighthouse Health and Wellness | - Increase happiness or well-being | - Information or education - Advice, tips, strategies, or skills training |
| Meditation Rx | - Increase happiness or well-being - Mindfulness, meditation, or relaxation - Reduce negative emotions | - Mindfulness or meditation - Relaxation |
| Mindarma | - Mindfulness, meditation, or relaxation | - Mindfulness or meditation |
| Mindfulness Coach | - Increase happiness or well-being - Reduce negative emotions - Depression - Anxiety or stress - Physical health | - Mindfulness or meditation |
| Mindshift | - Anxiety or stress | - CBT (behavioural) - CBT (cognitive) |
| MOVE! Coach | - Physical health | - Feedback - Information or education - Monitoring or tracking - Goal setting - Advice, tips, strategies, or skills training |
| OSI Connect | - Reduce negative emotions - Depression - Anxiety or stress - Anger | - Information or education - Monitoring or tracking - Advice, tips, strategies, or skills training |
| PeerConnect | - Increase happiness or well-being - Relationships | - Information or education - Advice, tips, strategies, or skills training |
| PE Coach 2 | - Other (PTSD) | - Feedback - Information or education - Monitoring tracking - Advice, tips, strategies, or skills training - Mindfulness or meditation - Relaxation - Other (prolonged exposure) |
| PTSD Coach | - Other (PTSD) | - Assessment - Information or education - Advice, tips, strategies, or skills training |
| PTSD Coach Canada | - Other (PTSD) | - Assessment - Information or education - Advice, tips, strategies, or skills training |
| PTSD Family Coach | - Other (PTSD) | - Assessment - Information/Education - Advice/Tips/Strategies/Skills training |
| R2MR | - Increase happiness or well-being - Behaviour change | - Information or education - Monitoring or tracking - Advice, tips, strategies, or skills training |
| Simply Yoga | - Mindfulness, meditation, or relaxation - Physical health | - Mindfulness or meditation - Relaxation |
| STAIR Coach | - Increase happiness or well-being - Reduce negative emotions - Relationships | - Information or education - Monitoring or tracking - Goal setting - Advice, tips, strategies, or skills training - CBT (behavioural) - CBT (cognitive) |
| Stand Down: Think Before You Drink | - Behaviour change - Alcohol or substance use | - Information or education - Monitoring or tracking - Goal setting - Advice, tips, strategies, or skills training |
| Stay Quit Coach | - Behaviour change - Alcohol or substance use - Other (PTSD) | - Information or education - Monitoring or tracking - Goal setting - Advice, tips, strategies, or skills training |
| Substance Abuse and Mental health Services Administration Disaster App | - Behaviour change | - Advice, tips, strategies, or skills training |
| SwapMyMood | - Increase happiness or well-being - Reduce negative emotions | - Information or education - Monitoring or tracking - Goal setting |
| VetChange | - Behaviour change - Alcohol or substance use | - Information or education - Monitoring or tracking - Goal setting - Advice, tips, strategies, or skills training |
| Virtual Hope Box | - Increase happiness or well-being - Mindfulness, meditation, or relaxation | - Information or education - Monitoring or tracking - Advice, tips, strategies, or skills training - Relaxation |
