# Supplementary material for: Digital Health Resilience and Well-Being Interventions for Military Members, Veterans, and Public Safety Personnel: Environmental Scan and Quality Review
Source: JMIR Mhealth Uhealth. 2025 Apr 1;13:e64098. doi: 10.2196/64098 (PMC12000787; doi:10.2196/64098)
Supplement: Multimedia Appendix 7 [file mhealth_v13i1e64098_app7.docx]

Mean A-MARS Ratings

| E-Tool Name | Engagement | Functionality | Aesthetic | Information | Quality | Subjective Quality | Health-  related Quality | Total |
| --- | --- | --- | --- | --- | --- | --- | --- | --- |
| AboutFace | 2.9 | 4.5 | 4.2 | 3.6 | 3.8 | 2.0 | 3.3 | 3.4 |
| AIMS for Anger Management^a^ | 3.6 | 4.8 | **4.3** | 4.1 | **4.2** | 3.9 | 4.3 | **4.1** |
| Chris Germer Meditations | 2.4 | 4.5 | *3.0* | 3.3 | 3.3 | 2.0 | 1.9 | 2.9 |
| Exalted Warrior Foundation | 2.4 | 3.9 | *3.0* | 2.5 | 2.9 | 1.9 | 1.8 | 2.6 |
| First Responders First | 2.8 | 3.9 | 3.3 | 3.2 | 3.3 | 2.5 | 4.0 | 3.3 |
| Freedom Qi Gong^a^ | 2.2 | 3.5 | 3.3 | 2.8 | 2.9 | 2.3 | 2.5 | 2.8 |
| Head to Health | 2.7 | 3.6 | 3.7 | 3.8 | 3.4 | 2.5 | 4.0 | 3.4 |
| HeadFIT^a^ | 3.4 | 4.5 | 4.2 | 3.8 | 4.0 | 3.0 | 3.5 | 3.7 |
| Manage Stress: VA National Center for Health Promotion and Disease Prevention | 3.2 | 4.3 | 3.7 | **4.8** | 4.0 | 3.1 | **4.7** | 4.0 |
| Meditation Oasis Podcasts | 2.4 | 4.3 | 3.7 | 2.8 | 3.3 | 1.9 | 2.8 | 3.0 |
| Mind Resilience Intervention | 2.6 | 3.8 | 4.0 | 4.0 | 3.6 | 2.5 | 4.2 | 3.5 |
| Misadventures in Money Management^a^ | **4.5** | 4.0 | 4.0 | 4.2 | **4.2** | **4.3** | 3.8 | **4.1** |
| National Sleep Foundation | 2.8 | 4.3 | 4.0 | 4.4 | 3.9 | 2.8 | 3.8 | 2.8 |
| NHS Every Mind Matters | 3.0 | 4.3 | 4.0 | 4.0 | 3.8 | 3.1 | 4.4 | 3.0 |
| Pain and Opioid Safety | 3.1 | 4.5 | 3.5 | 4.2 | 3.8 | 2.5 | 3.8 | 3.1 |
| Pain eHealth for Activity, Skills, and Education (resource section) | 2.9 | 3.8 | 3.5 | 3.9 | 3.5 | 2.6 | 3.4 | 2.9 |
| Provider Resilience^a^ | *2.1* | 3.1 | 3.2 | *1.7* | *2.5* | *1.8* | 2.5 | *2.4* |
| Responder Strong | 2.5 | *3.0* | *3.0* | 2.8 | 2.8 | *1.8* | 3.6 | 2.5 |
| Shield of Resilience Training^a^ | 3.4 | 4.6 | 3.8 | 4.0 | 4.0 | 2.4 | 2.9 | 3.4 |
| Tactical Breather | 2.5 | **4.9** | 3.3 | 3.5 | 3.6 | 2.4 | 1.7 | 2.5 |
| Tao Connect^a^ | 2.8 | *3.0* | 3.5 | 3.0 | 3.1 | 2.3 | 3.1 | 2.8 |
| Ten Percent Happier | 2.6 | 3.4 | 3.2 | 3.0 | 3.0 | 2.0 | 1.8 | 2.6 |
| VA Make the Connection | 3.4 | 4.0 | 4.0 | 4.2 | 3.9 | 3.0 | **4.7** | 3.4 |
| VA National Center for PTSD | 3.5 | 4.3 | 3.3 | 4.6 | 3.9 | 2.9 | 4.3 | 3.5 |
| VA Public Health | 3.5 | 4.3 | 3.8 | 3.6 | 3.8 | 2.5 | 3.3 | 3.5 |
| Veterans Yoga Project | 3.2 | 3.8 | 3.3 | 3.0 | 3.3 | 2.4 | *1.6* | 3.2 |
| Yoga Journal | 2.8 | 4.5 | 4.0 | 3.0 | 3.6 | 2.3 | 2.0 | 2.8 |

^a^ Web-based programs

Note: bolded text = highest score in that domain; italicized = lowest score in that domain
