# Supplementary material for: Digital Health Resilience and Well-Being Interventions for Military Members, Veterans, and Public Safety Personnel: Environmental Scan and Quality Review
Source: JMIR Mhealth Uhealth. 2025 Apr 1;13:e64098. doi: 10.2196/64098 (PMC12000787; doi:10.2196/64098)
Supplement: Multimedia Appendix 8 [file mhealth_v13i1e64098_app8.docx]

Mean ARIA Ratings

| **App Name** | **Part A** | **Part B** | **I would recommend using this app to the user** | **Stars that best represents the overall rating for the quality of this app** |
| --- | --- | --- | --- | --- |
| 7-Minute Chi | *21* | *36* | 3 | 2 |
| ACT Coach | 24.5 | 45 | 3.5 | 3 |
| AIMS for Anger Management | 24.5 | 44.5 | 4 | 4 |
| Breathe2Relax | 21 | 40 | 3.5 | 3 |
| Calm app | 22 | 42 | 3.5 | 3 |
| CBT-Insomnia Coach | 26 | 47 | 4 | 4 |
| Chill Drills | 24 | 43 | 4 | 3 |
| Comfort Talk Pro App | 25 | 43.5 | 4 | 3 |
| Couples Coach | 27 | 47.5 | 4 | 4 |
| COVID Coach | 25 | 46 | 4 | 4 |
| CPT Coach | 27 | 45.5 | 4 | 3.5 |
| CrewCare | 24.5 | 43 | 3.5 | 3 |
| Daily Yoga | 22 | 38.5 | 3 | 2 |
| Drinks:Ration | 24 | 42 | 3.5 | 2.5 |
| Driven Resilience App | 21.5 | 38 | 3 | 2.5 |
| equipt | 24 | 36 | 3 | 2 |
| eQuoo | 21.5 | 38 | 3 | 2.5 |
| FOCUS on the Go! | 23 | 42 | 4.5 | 4 |
| Insight Timer | 22.5 | 45 | 4 | 3 |
| Insomnia Coach | 26 | 45.5 | 4.5 | 3.5 |
| Lighthouse Health and Wellness | 23.5 | 39 | 3.5 | 2.5 |
| Meditation Rx | 27 | 38 | 3.5 | 3 |
| Mindarma | 22.5 | 44 | 3.5 | 3 |
| Mindfulness Coach | 25 | 46.5 | 4 | 4 |
| Mindshift App | 25 | 46.5 | 4 | 3 |
| MOVE! Coach | 26 | 37 | 3 | 2 |
| OSI Connect | 26.5 | 46 | 4 | 3 |
| PeerConnect App | 26.5 | 38.5 | *2.5* | *1.5* |
| PE Coach 2 | 26.5 | 40 | 3.5 | 2.5 |
| PTSD Coach | 25 | 49.5 | 4 | 3.5 |
| PTSD Coach Canada | 24 | 47 | 4 | 3.5 |
| PTSD Family Coach | 24.5 | **50** | 4.5 | 3.5 |
| R2MR | 25 | 48.5 | **5** | **4.5** |
| Simply Yoga | 21.5 | 44 | 3.5 | 3 |
| STAIR Coach | 25 | 46 | 4 | 3.5 |
| Stand Down: Think Before You Drink | 23.5 | 45.5 | 3.5 | 3 |
| Stay Quit Coach | 25.5 | 47 | 4 | 3 |
| Substance Abuse and Mental Health Services Administration’s Disaster | 23 | 37.5 | 3 | 2 |
| SwapMyMood | **29** | 44.5 | 3.5 | 3 |
| VetChange | 25 | 47.5 | 4 | 3.5 |
| Virtual Hope Box | 24.5 | 48 | 4 | 4 |

Note: Bolded text = highest score in that domain, and italicized text = lowest score in that domain
